# Postoperative Antibiotics Do Not Prevent Surgical Site Infections After Routine Ophthalmic Soft Tissue Surgeries in Dogs—A Retrospective Study

**DOI:** 10.1111/vop.70171

**Published:** 2026-04-03

**Authors:** Ana Cristina Piroth, Claudia Busse

**Affiliations:** ^1^ Department of Small Animal Medicine and Surgery University of Veterinary Medicine Hannover, Foundation Hannover Germany

**Keywords:** antimicrobial resistance, brachycephalic breeds, canine ophthalmology, Clavien–Dindo‐classification, risk factors, surgical complications

## Abstract

**Purpose:**

To compare the incidence of surgical site infections (SSI) following routine ophthalmic soft tissue surgeries in dogs receiving (AB) and not receiving (noAB) postoperative antibiotics.

**Methods:**

Clinical records of dogs that underwent enucleations, wedge resections, medial canthoplasties, and simple and complicated eyelid surgeries with at least 3 months follow‐up were retrospectively reviewed. SSI were recorded when increased mucoid or purulent discharge was present postoperatively or wound dehiscence occurred. SSI were graded based on Clavien–Dindo's classification according to the therapeutic intervention required.

**Results:**

A total of 231 dogs (AB = 108; noAB = 123) met the inclusion criteria for enucleations, 135 for wedge resections (AB = 62; noAB = 73), 31 for medial canthoplasties (AB = 15; noAB = 16), and 30 and 22 for simple (AB = 13; noAB = 17) and complicated (AB = 7; noAB = 15) eyelid surgeries, respectively. There was no significant difference in SSI rates between patients receiving topical or systemic postoperative antibiotics and those not receiving antibiotics. Variables associated with an increased risk of SSI included a brachycephalic phenotype in eyelid wedge resections and high body weight in simple and complicated eyelid surgeries. The SSI were classified as grade 1 in 6/32 (18.8%) patients and grade 2 in 20/32 (62.5%) patients. 4/32 (12.5%) patients with SSIs were classified as grade 3b and required surgical intervention.

**Conclusion:**

Postoperative systemic and topical antibiotics did not reduce SSI. The high incidence of SSI in medial canthoplasties and eyelid surgeries independent of postoperative antibiotic use highlights the need to reconsider pre‐, peri‐ and postoperative protocols, including suture material selection and the implementation of wound cleaning routines.

## 1 Introduction

Surgical site infections (SSI) are defined as infections occurring at the surgical site within 30 days postoperatively [1, 2]. SSI are categorized into incisional infections, which can be further subdivided into superficial and deep SSIs, and organ space infections [3]. In dogs, the reported incidence of SSIs varies widely depending on the performed procedure, ranging from 0.8% to 18.1% in small animal surgeries [4, 5]. The primary pathogens involved are gram‐positive cocci, particularly staphylococci, which are notable for their antibiotic resistance potential [6, 7]. SSI represent a considerable challenge in veterinary practice, as they are associated with increased morbidity, elevated treatment costs, and prolonged recovery periods [5, 8]. Antibiotics are therefore frequently administered postoperatively to prevent SSI, a practice that is becoming increasingly disputable [9, 10].

In human medicine, studies on ophthalmic soft tissue surgeries have raised questions about the necessity of this practice. Evidence suggests that perioperative intravenous and postoperative oral antibiotics do not reduce SSI rates after routine enucleations or eviscerations with implants [11]. Furthermore, the routine use of systemic antibiotics after elective eyelid surgery did not significantly impact infection rates [12]. Similarly, in veterinary medicine, recent data do not support the routine use of prophylactic antibiotics following enucleation in dogs [13]. Despite this, a recent online survey among German veterinarians revealed that approximately two‐thirds of participants routinely prescribed postoperative antibiotics following ophthalmic soft tissue procedures [10]. Given the global concern about antimicrobial resistance, this discrepancy is noteworthy and raises essential questions about the true benefit of postoperative antibiotic use.

This study was therefore designed to assess the incidence of SSIs in dogs undergoing different routine ophthalmic soft tissue surgeries, with and without postoperative antibiotic treatment.

## 2 Materials and Methods

### 2.1 Animals and Inclusion Criteria

A retrospective review of medical records was conducted. Inclusion criteria consisted of dogs that underwent routine enucleations, eyelid wedge resections, medial canthoplasties, and both simple and complicated eyelid surgeries at the Clinic for Small Animals, University of Veterinary Medicine Hannover, Foundation between January 2017 and September 2024.

Risk factors were categorized as patient‐related, surgery‐related, and antibiotic‐related factors. Each patient's age, weight, breed, and cephalic conformation were recorded. Dogs were classified as brachycephalic based on the breed classification suggested by Packer et al. [14]. Phenotypic characteristics were assessed, including the presence of giant breeds weighing more than 40 kg, dogs with pronounced breed‐related skin folds (including Shar Pei and Chow Chow), toy breeds weighing less than 2 kg, and puppies younger than 3 months. Prior to the medical data search, co‐morbidities with a known or presumed impact on wound healing were predefined and specifically assessed. These included diabetes mellitus; hypothyroidism; Cushing's disease; dermatological diseases associated with skin lesions; allergies (food‐related or environmental); non‐ocular neoplasia; and immune‐mediated diseases under medical treatment [15, 16, 17, 18, 19]. Conditions that did not fall into these categories and had no known impact on wound healing were marked as “other.” Glucocorticoid administration was categorized as either long‐term use, defined as continuous administration without a predefined endpoint, or recent use, defined as short‐term administration with a planned tapering and discontinuation. The indication for surgery and the surgical approach were recorded. If additional ophthalmic or non‐ophthalmic procedures were performed, they were recorded. The surgeon performing each procedure was recorded and categorized as an ECVO or ECVS diplomate, an ECVO resident, or an assistant veterinarian.

For all surgeries, different combinations of suture materials were compared, including absorbable monofilament (e.g., polydioxanone, poliglecapron 25), non‐absorbable monofilament (e.g., nylon), and absorbable braided multifilament sutures (e.g., polyglactin 910); in enucleation procedures, the use of orbital mesh as well as the application of a skin fold cover suture were recorded. The use of perioperative and postoperative antibiotics was assessed (yes/no), and when administered, the active ingredient was recorded; postoperative antibiotic use was additionally classified as systemic or topical. For enucleations, SSI were defined in the presence of purulent wound secretion, wound dehiscence, excessive swelling, or delayed hematoma formation. For eyelid wedge resections, medial canthoplasties, and simple or complicated eyelid surgeries, SSI were defined by excessive mucoid or purulent wound secretion and/or wound dehiscence. In all surgical procedures, cases were additionally classified as SSI when the attending or referring veterinarian prescribed antibiotics during follow‐up examinations due to suspected infection, even if the specific clinical basis for this suspicion was not reported.

In cases where an SSI occurred, the initiation, modification, or continuation of topical and/or systemic antibiotics or antiseptics, as well as any wound debridement under local anesthesia or surgical revision under general anesthesia, were recorded. The Clavien–Dindo classification of surgical complications was used to describe the severity of SSIs based on the therapeutic interventions required [20] (Table 1). Bacterial culture results were recorded when sampling was performed in cases of SSI. Culture results were evaluated in the context of the overall clinical presentation. A negative culture result alone was not considered sufficient to rule out an SSI when clinical findings were clearly indicative of infection. Only patients with a follow‐up of at least 3 months were included, based on re‐examinations or telephone contact with the owner or the local veterinarian.

**TABLE 1:** Clavien–Dindo classification of surgical complications [20].

Grades	Definition
Grade I	Any deviation from the normal postoperative course without the need for pharmacological treatment, or surgical, endoscopic, and radiological interventions Allowed therapeutic regimens are: Drugs as antiemetics, antipyretics, analgetics, diuretics and electrolytes, and physiotherapy. This grade also includes wound infections opened at the bedside
Grade II	Requiring pharmacological treatment with drugs other than such allowed for grade I complications Blood transfusions and total parenteral nutrition are also included
Grade III	Requiring surgical, endoscopic, or radiological intervention
Grade IIIa	Intervention not under general anesthesia
Grade IIIb	Intervention under general anesthesia
Grade IV	Life‐threatening complication (including central nervous system complications) requiring IC/ICU management
Grade IVa	Single organ dysfunction (including dialysis)
Grade IVb	Multiorgan dysfunction
Grade V	Death of a patient

*Enucleations* were performed by either a transpalpebral or subconjunctival approach or by exenteration. For the purpose of this study, the term “enucleation” was used as a collective category including both enucleations and orbital exenterations. Only a small number of exenterations were included. Although exenterations represent a more extensive surgical procedure involving removal of the orbital contents, they were grouped together with enucleations for analytical purposes and to improve overall readability. The ophthalmic findings necessitating enucleation or exenteration were classified as either infectious (e.g., ulcerative keratitis or corneal perforation with presumed bacterial infection), non‐infectious (e.g., glaucoma), or neoplastic (e.g., intra‐ or extraocular neoplasia). Dogs that presented with severe head trauma and associated periorbital injuries or orbital fractures requiring skin flaps or grafting techniques for proper closure or wound drainage were excluded from the study.

*Eyelid wedge resections* were closed using a single‐ or two‐layered wound closure with a figure‐of‐eight suture pattern and the remaining wound by single, interrupted sutures as necessary. Additional tarsorrhaphy for tension relief was recorded when performed. Eyelid lacerations were only included in chronic cases where eyelid reconstruction was performed after secondary wound granulation and infection control. The exact type of neoplasia or inflammatory lesion was confirmed only in cases where the resected sample was submitted for histopathologic examination.

*Medial canthoplasties* were performed by removing a portion of the overly eyelid fissure length and the nasal caruncle to shorten the palpebral fissure. The lacrimal canaliculi were split longitudinally to allow opening of the lacrimal drainage system. Closure was routinely performed using a figure‐of‐eight suture pattern, followed by a two‐layered wound closure. The procedure was carried out in accordance with the technique described by Allgoewer et al. [21].

*Eyelid surgeries* were categorized as simple or complicated procedures. Simple corrections were performed using the Celsus‐Hotz procedure alone or simple ectropion corrections that only included one surgical incision line. In comparison, complicated cases required more than one surgical incision line, including the modified Kuhnt–Szymanowski procedure combined with Celsus‐Hotz, the arrowhead procedure, the Gutbrod and Tietz technique, Stades' forced granulation method for upper eyelid trichiasis and blepharoplasties using combinations of different surgical approaches.

### 2.2 Statistical Analysis

Statistical analyses were performed with SAS (SAS Institute Inc., 2002–2020, Cary, NC, USA). Categorical variables were summarized as absolute numbers and percentages. Associations between potential risk factors and the occurrence of SSI were evaluated using two‐sided Fisher's exact tests. For variables with two categories, 2 × 2 contingency tables were constructed. For variables with more than two categories, each category was compared against the remaining population using separate 2 × 2 contingency tables (category vs. rest approach). Due to small subgroup sizes and low expected cell counts, chi‐square testing was not applied. Multivariable analysis was not performed because of the limited sample size and low number of SSI events. A *p*‐value < 0.05 was considered statistically significant.

## 3 Results

Across the five surgical procedures, enucleations comprised 231 observations with five SSIs (2.2%) and 40 dogs excluded due to insufficient follow‐up; eyelid wedge resections included 135 observations with nine SSIs (6.7%) and 22 excluded dogs; medial canthoplasties were represented by 31 observations in which five SSIs (16.1%) occurred and five dogs were excluded; simple eyelid surgeries accounted for 30 observations with four SSIs (13.3%) and three exclusions; and complicated eyelid surgeries involved 22 observations with nine SSIs (41%) and seven dogs excluded due to limited follow‐up.

### 3.1 Enucleations

#### 3.1.1 Patient‐Related Risk Factors

A total of 231 dogs met the inclusion criteria. The mean age of the dogs was 8.4 ± 4.1 years (range 0–18 years; *n* = 231). The primary indication for enucleation was non‐infectious disease (168/231; 73%), followed by infectious disease of the ocular surface (42/231; 18%) and neoplastic diseases (21/231; 9%). All investigated patient‐related risk factors, including age, weight, specific phenotypes, co‐morbidities and the influence of immunosuppression showed no significant association with SSI (Table 2).

**TABLE 2:** Risk factors for SSI after enucleations.

Enucleations (*n* = 231)				
Variables	Total (*n*)	SSI (*n*)	SSI (%)	*p*
*Weight (kg)*				
< 10	97	2	2.1	1.00*
10–20	68	0	0	0.33*
20–30	33	1	3.0	0.54*
> 30	33	2	6.1	0.15*
*Phenotype*				
None	173	5	2.9	0.33*
Brachycephalic	52	0	0	0.59*
Giant breed (> 40 kg)	2	0	0	1.00*
Heavy breed related folds	1	0	0	1.00*
Toy breeds (< 2 kg)	6	0	0	1.00*
Neonates (< 3 months of age)	1	0	0	1.00*
*Co‐morbidities*				
None	69	1	1.4	0.62*
Diabetes mellitus	5	1	20.0	0.18*
Hypothyreoidism	7	1	14.3	0.23*
Cushing's disease	2	0	0	1.00*
Skin lesions	12	0	0	1.00*
Allergy (Food, environmental)	10	0	0	1.00*
Non‐ocular tumor	15	2	13.3	0.11*
Immunemediated disease	7	0	0	1.00*
*Immunosuppression*				
Yes	17	0	0	
No	214	5	2.3	0.68*
*Indication für surgery*				
Infectious	42	1	2.4	1.00*
Non‐infectious	168	3	1.8	0.62*
Neoplastic	21	1	4.8	0.38*
*Surgical approach*				
Subconjunctival	39	0	0	0.59*
Transpalpebral	187	5	2.7	0.59*
Exenteration	3	0	0	1.00*
*Surgeon group*				
Diplomate ECVO/ECVS	24	1	4.2	0.43*
Resident ECVO	114	2	1.8	1.00*
Assistant	93	2	2.2	1.00*
*Perioperative antibiotics*				
Yes	206	4	1.9	
No	25	1	4.0	0.44*
*Perioperative antibiotics—agents*				
None	25	1	4.0	0.44*
Cefazolin/Cephalexin i.v. (1st‐generation cephalosporin) i.v.	48	2	4.2	0.27*
Cefuroxime i.v. (2nd‐generation cephalosporin) i.v.	91	0	0	0.16*
Amoxicillin–clavulanic acid i.v.	10	1	10.0	0.20*
Amoxicillin–clavulanic acid (175 mg/mL) s.c.	48	1	2.1	1.00*
Enrofloxacin i.v.	1	0	0	1.00*
Doxycycline	2	0	0	1.00*
Preoperative oral or i.v. antibiotic therapy without perioperative administration	6	0	0	1.00*
Combination of two different antibiotics	2	0	0	1.00*
*Postoperative antibiotics*				
Yes	108	3	2.8	
No	123	2	1.6	0.67*
*Postoperative antibiotics—agents*				
None	123	2	1.63	0.67*
Cefazolin/Cephalexin (1st‐generation cephalosporin) p.o.	17	0	0	1.00*
Amoxicillin–clavulanic acid p.o.	80	3	3.6	0.33*
Fluoroquinolone (enrofloxacin, marbofloxacin, pradofloxacin) p.o.	4	0	0	1.00*
Docycycline p.o.	5	0	0	1.00*
Trimethoprim–sulfonamide p.o.	1	0	0	1.00*
Metronidazole p.o.	1	0	0	1.00*
*Suture material for skin closure*				
Absorbable monofilament	151	3	2.0	0.72*
Non‐absorbable monofilament	80	2	2.5	1.00*
Orbital mesh	79	3	3.8	0.43*

*Note:* n/a from medical records; *p* < 0.05 is considered significant.

Abbreviations: i.v., intravenously; n/a, not applicable; p.o., per oral; s.c., subcutaneously.

* Fisher's exact test (2 × 2).

#### 3.1.2 Surgery‐Related Risk Factors

The transpalpebral approach was used in 187/231 (81%) cases, the subconjunctival approach in 39/231 (17%), and exenteration in 3/231 (1%). Surgeries were performed by ECVO/ECVS diplomates in 24/231 (10%) dogs, by ECVO residents in 114/231 (49%), and by assistant veterinarians in 93/231 (40%). The surgical approach, the surgeon group, and suture material used for wound closure were not associated with a significant risk for developing SSI (Table 2).

#### 3.1.3 Antibiotic‐Related Risk Factors

**FIGURE 1:**
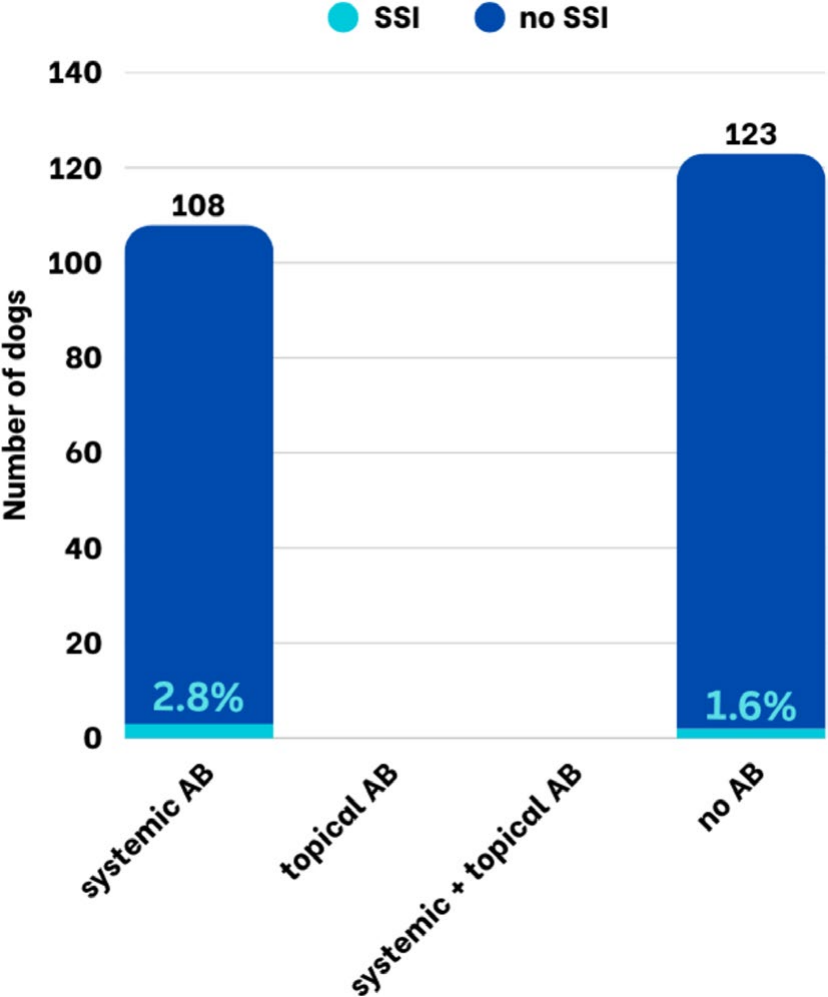
Proportion of dogs with and without SSI after enucleations considering different postoperative antibiotic regimens.

Postoperative antibiotics were administered to 108/231 (47%) patients, while 123/231 (53%) did not receive antibiotics. SSI occurred in 3/108 dogs receiving postoperative antibiotics (2.8%) and in 2/123 dogs without postoperative antibiotics (1.6%) (Figure 1)—this difference was not statistically significant. The use of perioperative antibiotics, including the specific agents administered, was not significantly associated with the occurrence of SSIs (Table 2).

#### 3.1.4 Surgical Site Infections (SSI)

Overall, SSI occurred in 5/231 (2.2%) dogs (Table 7). According to the Clavien–Dindo classification, 3/5 (60%) were classified as grade 1. Within 3 months postoperatively, surgical site swelling was observed in all cases, with hematoma development additionally noted in one case. These cases were managed conservatively with a wait‐and‐see approach. One case was classified as grade 2 and required systemic antibiotics, and one case was classified as grade 3b and required surgical revision. Information for the grade 2 and grade 3 cases was obtained by telephone contact with the owner.

### 3.2 Eyelid Wedge Resections

#### 3.2.1 Patient‐Related Risk Factors

A total of 135 dogs met the inclusion criteria. The mean age of the dogs was 9.3 ± 2.9 years (range 2–18 years; *n* = 135). The primary indication for wedge resection was eyelid tumors (131/135; 97%). Of these, 109/131 (83%) were histopathologically confirmed as benign, while 22/131 (17%) were not submitted for histopathological examination at the owners' request. Non‐neoplastic indications were rare and included distichiasis/trichiasis/ectopic cilia (2/135; 1%), wedge resection following traumatic eyelid laceration (1/135; 1%), and one eyelid dermoid (1/135; 1%). Fisher's exact test revealed a significantly higher complication rate in brachycephalic dogs and a significantly lower rate in dogs without phenotypic risk factors, whereas no significant associations were found for giant breeds, heavy breed‐related folds, or toy breeds (Table 3). All other investigated patient‐related risk factors, including age, weight, co‐morbidities, and the influence of immunosuppression showed no significant association with SSI (Table 3).

**TABLE 3:** Risk factors for SSI after wedge resections.

Eyelid wedge resections (*n* = 135)				
Variables	Total (*n*)	SSI (*n*)	SSI (%)	*p*
*Weight (kg)*				
< 10	31	3	9.8	0.43*
10–20	26	2	7.7	0.69*
20–30	39	3	7.8	0.72*
> 30	39	1	2.6	0.45*
*Phenotype*				
None	111	4	3.6	**0.009** *
Brachycephalic	17	5	29.4	**0.002** *
Giant breed (> 40 kg)	3	0	0	1.00*
Heavy breed related folds	2	0	0	1.00*
Toy breeds (< 2 kg)	2	0	0	1.00*
*Co‐morbidities*				
None	33	2	6.1	1.00*
Diabetes mellitus	2	0	0	1.00*
Hypothyreoidism	2	0	0	1.00*
Cushing's disease	1	0	0	1.00*
Skin lesions	13	1	7.7	1.00*
Allergy (Food, environmental)	4	0	0	1.00*
Non‐ocular tumor	14	1	7.1	1.00*
Other (non‐wound‐healing related)	83	6	7.2	1.00*
*Immunosuppression*				
Yes	6	0	0	
No	129	9	7.0	1.00*
*Indication für surgery*				
Eyelid tumor				
Benign	109	8	7.3	1.00*
Not submitted for histopathology	22	0	0	0.35*
Ciliary abnormalities (distichiasis, ectopic cilia, trichiasis)	2	1	50	0.13*
Eyelid laceration	1	0	0	1.00*
Eyelid dermoid	1	0	0	1.00*
*Surgical approach*				
Eyelid wedge resection, figure of eight suture + additional skin closure (single‐ or double layer)	127	9	7.1	1.00*
Eyelid wedge resection, figure of eight suture + additional skin closure (single‐ or double layer) + temporary tarsorrhapy	5	0	0	1.00*
Not documented	3	0	0	1.00*
*Surgeon group*				
Diplomate ECVO	13	0	0	0.60*
Resident ECVO	65	6	9.2	0.31*
Assistant	57	3	5.3	0.73*
*Perioperative antibiotics*				
Yes	97	7	7.2	
No	38	2	5.3	1.00*
*Perioperative antibiotics—agents*				
None	38	2	5.3	1.00*
Cefazolin/Cephalexin i.v. (1st‐generation cephalosporin) i.v.	41	2	4.9	0.72*
Cefuroxime i.v. (2nd‐generation cephalosporin) i.v.	48	5	10.4	0.28*
Amoxicillin–clavulanic acid i.v.	1	0	0	1.00*
Amoxicillin–clavulanic acid (175 mg/mL) s.c.	6	0	0	1.00*
Doxycycline	1	0	0	1.00*
*Postoperative antibiotics*				
No antibiotics	73	5	6.8	1.00*
Systemic antibiotics	11	2	18.2	0.16*
Topical antibiotics	37	0	0	0.06*
Combined systemic and topical antibiotics	14	2	14.3	0.23*
*Postoperative antibiotics—agents*				
None	73	5	6.8	1.00*
Cefazolin/Cephalexin (1st‐generation cephalosporin) p.o.	10	2	20.0	0.16*
Amoxicillin–clavulanic acid p.o.	14	2	14.3	0.27*
Fluoroquinolone (enrofloxacin, marbofloxacin, pradofloxacin) p.o.	1	0	0	1.00*
Topical antibiotics	52	2	3.8	0.33*
Fusidic acid	10	0	0	1.00*
Chloramphenicol	41	2	4.9	1.00*
Ofloxacin	1	0	0	1.00*
*Suture material for skin closure*				
Absorbable braided	132	9	4.5	
Non‐absorbable monofilament	2	0	0	1.00*

*Note:* n/a from medical records; *p* < 0.05 is considered significant; bold numbers indicate statistical significance (*p* < 0.005).

Abbreviations: i.v., intravenously; n/a, not applicable; p.o., per oral; s.c., subcutaneously.

* Fisher's exact test (2 × 2).

#### 3.2.2 Surgery‐Related Risk Factors

The standard eyelid wedge resection with figure‐of‐eight closure was performed in 127/135 (94%) cases. This approach was combined with additional tarsorrhaphy in 5/135 (4%) cases; in 3/135 (2%), the surgical method was not specified. Surgeries were performed by ECVO/ECVS diplomates in 13/135 (10%) dogs, by ECVO residents in 65/135 (48%), and by assistant veterinarians in 62/135 (46%). The surgical approach, the surgeon group, and suture material used for wound closure were not associated with a significant risk for developing SSI (Table 3).

#### 3.2.3 Antibiotic‐Related Risk Factors

**FIGURE 2:**
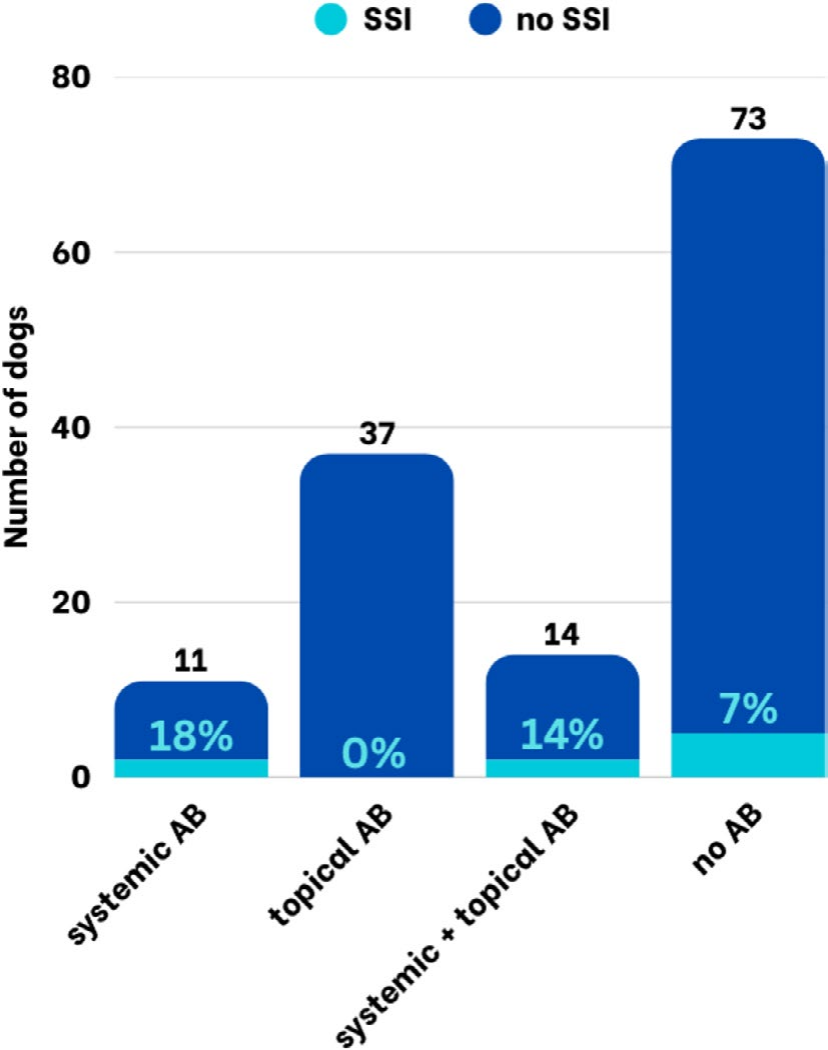
Proportion of dogs with and without SSI after wedge resections considering different postoperative antibiotic regimens.

Postoperative antibiotics were administered to 64/135 (47%) patients, while 73/135 (54%) did not receive antibiotics. SSI occurred in 4/62 dogs receiving postoperative antibiotics (6.5%) and in 5/73 dogs without postoperative antibiotics (6.8%) (Table 3). The difference was not statistically significant. When looking at the different antibiotic groups, the SSI rate for patients treated with systemic antibiotics, topical antibiotics, a combination of systemic and topical antibiotics, and without antibiotics was 18% (2/11), 0% (0/37), 14% (2/14), and 7% (5/73), respectively (Figure 2). When the different antibiotic groups were compared to the patients that did not receive antibiotics, the difference was not significant for any group. The use of perioperative antibiotics, including the specific agents administered, was not significantly associated with the occurrence of SSIs (Table 3).

#### 3.2.4 Surgical Site Infections (SSI)

**FIGURE 3:**
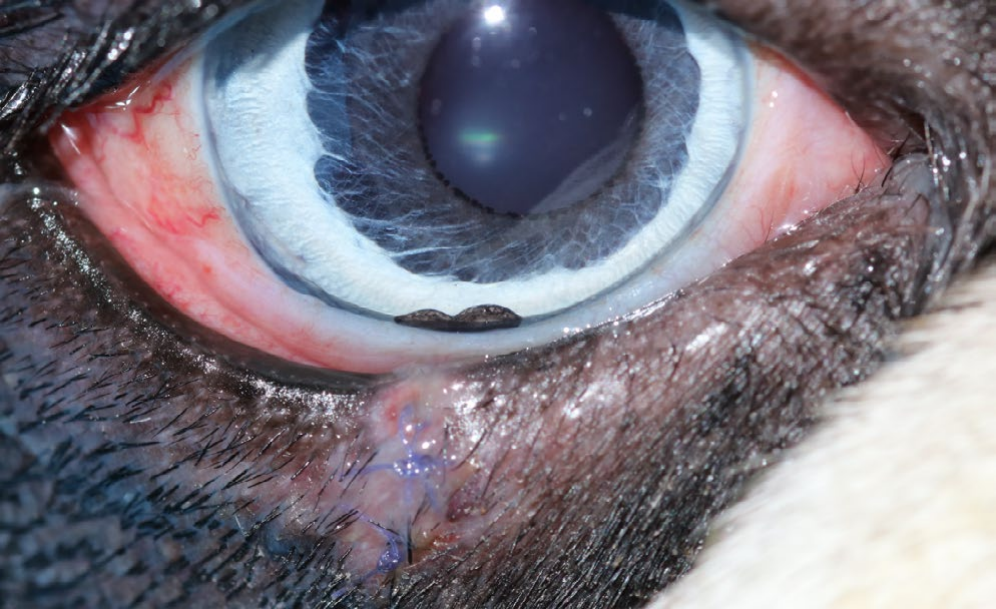
Right eye of a Boston Terrier with moderate mucoid discharge and beginning loosening of the sutures, indicating early wound dehiscence 7 days after wedge resection of a benign eyelid mass. A topical antiseptic was initiated, and the case was classified as grade 2 according to Clavien–Dindo's classification of surgical complications [20].

Overall, SSI occurred in 9/135 (6.7%) dogs (Table 7). Clinical signs included purulent ocular discharge (*n* = 1), wound dehiscence (*n* = 2), a combination of purulent discharge and wound dehiscence (*n* = 1), and mucoid discharge with wound dehiscence (*n* = 1) (Figure 3). According to the Clavien–Dindo classification, 7 cases were classified as grade 2 and required a change in medical treatment. One case was classified as grade 3b and required surgical revision. In one case, the exact grade could not be determined because information was obtained only through a telephone contact with the owner. In two cases, bacteriological examination using conventional bacterial culture following swab sample collection identified bacteria belonging to the families *Streptococcaceae*, *Pasteurellaceae*, and *Neisseriaceae*.

### 3.3 Medial Canthoplasties

#### 3.3.1 Patient‐Related Risk Factors

A total of 31 dogs met the inclusion criteria. The mean age of the dogs was 5.3 ± 3.0 years (median 6 years, range 0–18 years; *n* = 135). A brachycephalic phenotype was present in 28/31 (90%) dogs, while 3/31 (10%) were non‐brachycephalic. The most common breeds were Pugs (20/31; 65%) and Shih Tzus (4/31; 13%), followed by Malteses (2/31; 6%). Single cases included a Japanese Chin, a Bolonka Zwetna, and one mixed‐breed dog (each 3%). The primary indication for surgery in this group was macroblepharon, either alone or in combination with other conditions (25/31; 81%), followed by trichiasis (5/31; 16%) and pigmentary keratitis (7/31; 23%). All investigated patient‐related risk factors, including age, weight, specific phenotypes, co‐morbidities and the influence of immunosuppression, showed no significant association with SSI (Table 4).

**TABLE 4:** Risk factors for SSI after medial canthoplasties.

Medial canthoplasties (*n* = 31)				
Variables	Total (*n*)	SSI (*n*)	SSI (%)	*p*
*Weight (kg)*				
< 10	22	3	13.6	
10–20	9	2	22.2	0.61*
*Phenotype*				
None	3	0	0	
Brachycephalic	28	5	17.9	0.55*
*Co‐morbidities*				
None	14	4	28.6	0.15*
Skin lesions	3	0	0	1.00*
Allergy (food, environmental)	1	0	0	1.00*
Other (non‐wound‐healing related)	14	1	7.1	0.65*
*Immunosuppression*				
Yes	1	0	0	
No	30	5	16.7	1.00*
Indication für surgery				
Entropion	11	1	9.1	1.00*
Macroblepharon	25	5	20.0	0.419*
Trichiasis	5	1	20.0	0.562*
Pigmentary keratitis	7	0	0	0.573*
*Surgeon group*				
Diplomate ECVO	7	0	0	0.559*
Resident ECVO	19	4	21.1	0.634*
Assistant	5	1	20.0	1.000*
*Perioperative antibiotics*				
Yes	28	5	17.9	
No	3	0	0	1.00*
*Perioperative antibiotics—agents*				
None	3	0	0	1.00*
Cefazolin/Cephalexin i.v. (1st‐generation cephalosporin) i.v.	13	2	15.4	1.00*
Cefuroxime i.v. (2nd‐generation cephalosporin) i.v.	13	3	23.1	0.625*
Amoxicillin–clavulanic acid i.v.	1	0	0	1.00*
Amoxicillin–clavulanic acid (175 mg/mL) s.c.	1	0	0	1.00*
*Postoperative antibiotics*				
No antibiotics	16	4	25.0	0.33*
Systemic antibiotics	2	0	0	1.00*
Topical antibiotics	10	1	10.0	1.00*
Combined systemic and topical antibiotics	3	0	0	1.00*
*Postoperative antibiotics—agents*				
None	16	4	25.0	0.16*
Cefazolin/Cephalexin (1st‐generation cephalosporin) p.o.	3	0	0	1.00*
Amoxicillin–clavulanic acid p.o.	2	0	0	1.00*
Topical antibiotics	13	1	7.7	1.00*
Fusidic acid	2	0	0	1.00*
Chloramphenicol	11	1	9.1	1.00*
*Suture material for skin closure*				
Absorbable braided	31	5	16.1	

*Note:* n/a from medical records; *p* < 0.05 is considered significant.

Abbreviations: i.v., intravenously; n/a, not applicable; p.o., per oral; s.c., subcutaneously.

* Fisher's exact test (2 × 2).

#### 3.3.2 Surgery‐Related Risk Factors

All dogs underwent standard medial canthoplasty by removing a part of the medial canthal region and closure using a figure‐of‐eight suture pattern, followed by a two‐layered wound closure (31/31, 100%). Surgeries were performed by an ECVO diplomate in 7/31 (23%) dogs, by ECVO residents in 19/31 (61%), and by assistant veterinarians in 5/31 (16%). The surgical approach and the surgeon group were not associated with a significant risk for developing SSI (Table 4). Only absorbable braided suture material was used for skin closure; therefore, no statistical comparison was performed.

#### 3.3.3 Antibiotic‐Related Risk Factors

**FIGURE 4:**
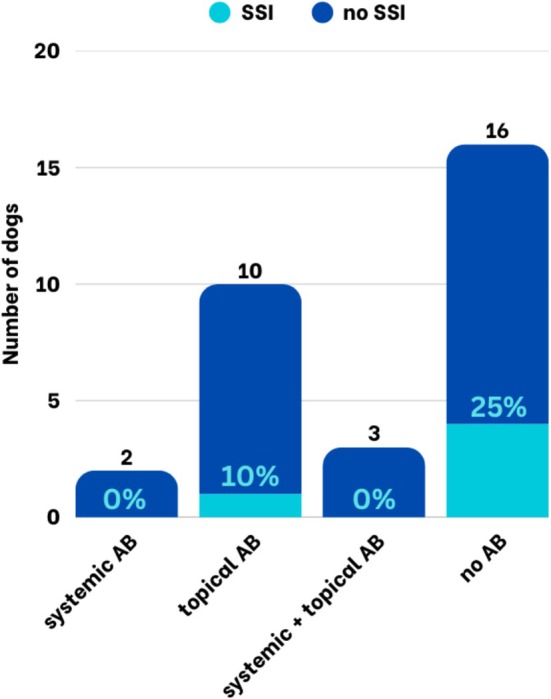
Proportion of dogs with and without SSI after medial canthoplasties considering different postoperative antibiotic regimens.

Postoperative antibiotics were administered to 15/31 (48%) patients, while 16/31 (52%) did not receive antibiotics. In dogs undergoing medial canthoplasty, the use of postoperative antibiotics had no significant effect on the occurrence of SSI. SSI occurred in 1/15 dogs receiving postoperative antibiotics (6.7%) and in 4/16 dogs without postoperative antibiotics (25.0%), but this difference was not statistically significant. When differentiated by postoperative antibiotic regimen, the SSI rate was 0% in dogs receiving systemic antibiotics, 10% in those treated with topical antibiotics, 0% in those receiving a combination of systemic and topical antibiotics, and 25% in dogs without postoperative antibiotics (Figure 4) – the difference was not significant (Table 4). The use of perioperative antibiotics, including the specific agents administered, was not significantly associated with the occurrence of SSIs (Table 4).

#### 3.3.4 Surgical Site Infections (SSI)

**FIGURE 5:**
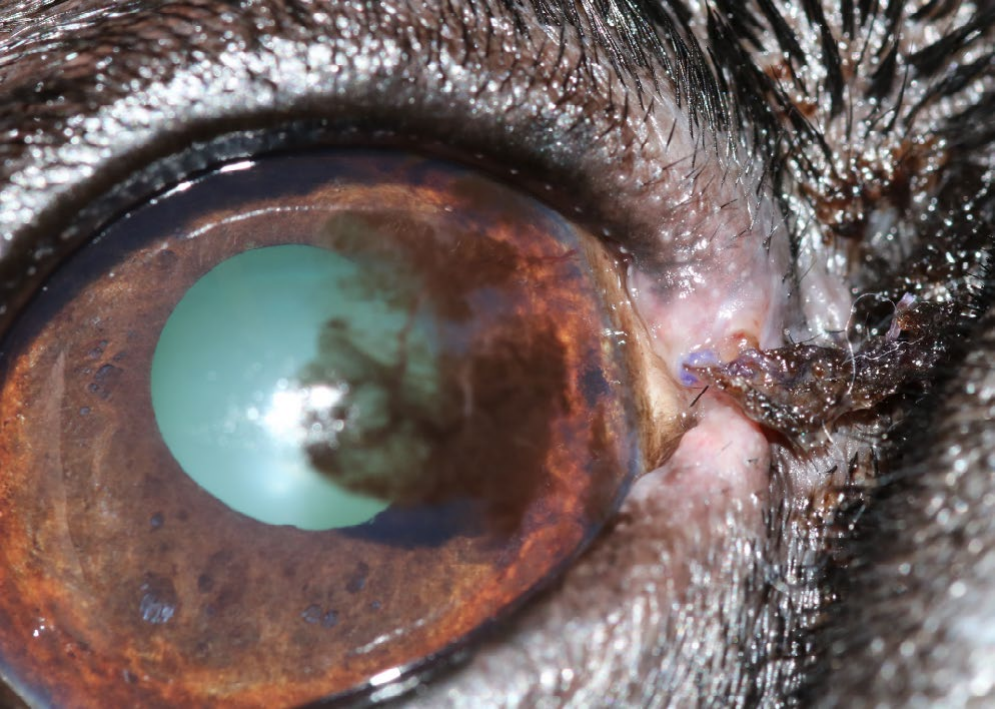
Right eye of a French Bulldog 6 days after medial canthoplasty for treating macroblepharon and pigmentary keratitis with mild mucoid discharge and wound dehiscence of the medial canthal area. This dog required surgical intervention under general anasthesia and was classified as grade 3b according to Clavien Dindo's classification of surgical complications [20].

Overall, SSI occurred in 5/31 (16.1%) dogs (Table 7). Complications included purulent wound secretion (*n* = 2), wound dehiscence (*n* = 4), and mucoid discharge (*n* = 1), with some dogs presenting more than one complication. The severity of SSIs was subsequently described using the Clavien–Dindo classification of surgical complications. According to this classification, two cases were graded as grade 1, in which no change in treatment was required and a wait‐and‐see approach was taken; one case was graded as grade 2, requiring a change in medical treatment; and two cases were graded as grade 3b, requiring surgical revision due to wound dehiscence (Figure 5). In one patient, microbiological examination of purulent discharge revealed gram‐positive cocci (Staphylococci spp., Streptococci spp.).

### 3.4 Simple Eyelid Surgeries

#### 3.4.1 Patient‐Related Risk Factors

A total of 30 dogs met the inclusion criteria. The mean age of the dogs was 1.6 ± 1.6 years (median 1 year, range 0–9 years; *n* = 30). Of the 30 dogs, 4 (13%) were classified as giant breeds, and 3/30 (10%) had breed‐related heavy skin folds. The most common breeds were mixed‐breed dogs (4/30; 13%), Labrador Retrievers (3/30; 10%), and German Wirehaired Pointers (3/30; 10%). Rhodesian Ridgebacks and Shar Peis were represented twice each (2/30; 7%). Dogs weighing > 30 kg had a significantly higher risk of SSI compared to dogs ≤ 30 kg. The primary indication for surgery in this group was entropion (26/30; 87%), followed by ectropion (4/30; 13%). All other investigated patient‐related risk factors, including age, specific phenotypes, co‐morbidities and the influence of immunosuppression showed no significant association with SSI (Table 5).

**TABLE 5:** Risk factors for SSI after simple eyelid surgeries.

Simple eyelid surgeries (*n* = 30)				
Variables	Total (*n*)	SSI (*n*)	SSI (%)	*p*
*Weight (kg)*				
< 10	2	0	0	1.00*
10–20	5	0	0	1.00*
20–30	10	0	0	0.27*
> 30	13	4	30.8	**0.026** *
*Phenotype*				
None	23	3	13.0	1.00*
Giant breed (> 40 kg)	4	1	25.0	0.51*
Heavy breed related folds	3	0	0	1.00*
*Co‐morbidities*				
None	16	3	18.8	0.30*
Hypothyreoidism	1	0	0	1.00*
Skin lesions	3	0	0	1.00*
Allergy (food, environmental)	1	0	0	1.00*
Other (non‐wound‐healing related)	11	1	9.1	1.00*
*Immunosuppression*				
Yes	1	0	0	
No	29	4	13.8	1.00*
*Indication for surgery*				
Entropion	26	3	11.5	
Ectropion	4	1	25	0.45*
*Surgical approach*				
Celsus‐Hotz procedure	26	2	7.7	
V‐Y‐Plasty	4	2	50.0	**0.04** *
*Surgeon group*				
Diplomate ECVO	7	2	28.6	0.21*
Resident ECVO	11	1	9.1	0.63*
Assistant	12	1	8.3	1.00*
*Perioperative antibiotics*				
Yes	21	3	14.3	
No	9	1	11.1	1.00*
*Perioperative antibiotics—agents*				
None	9	1	11.1	1.00*
Cefazolin/Cephalexin i.v. (1st‐generation cephalosporin) i.v.	11	2	18.2	0.60*
Cefuroxime i.v. (2nd‐generation cephalosporin) i.v.	7	1	14.3	1.00*
Amoxicillin–clavulanic acid (175 mg/mL) s.c.	3	0	0	1.00*
*Postoperative antibiotics*				
No antibiotics	17	3	17.6	0.32*
Systemic antibiotics	2	0	0	1.00*
Topical antibiotics	8	1	12.5	1.00*
Combined systemic and topical antibiotics	3	0	0	1.00*
*Postoperative antibiotics—agents*				
None	17	3	17.6	0.32*
Cefazolin/Cephalexin (1st‐generation cephalosporin) p.o.	2	0	0	1.00*
Amoxicillin–clavulanic acid p.o.	3	0	0	1.00*
Doxycycline p.o.	1	0	0	1.00*
Topical antibiotics	11	1	9.1	1.00*
Chloramphenicol	11	1	9.1	1.00*
*Suture material for skin closure*				
Absorbable braided	30	4	13.3	

*Note:* n/a from medical records; *p* < 0.05 is considered significant; bold numbers indicate statistical significance (*p* < 0.005).

Abbreviations: i.v., intravenously; n/a, not applicable; p.o., per oral; s.c., subcutaneously.

* Fisher's exact test (2 × 2).

#### 3.4.2 Surgery‐Related Risk Factors

The vast majority of dogs underwent a Hotz–Celsus procedure (26/30; 87%), while a V–Y plasty was performed in 4/30 (13%) of dogs in this group. A significantly higher incidence of SSI was observed following V–Y plasty (50.0%) compared to the Celsus–Hotz procedure (7.7%) (Table 5). However, this finding should be interpreted with caution due to the small sample size in the V–Y group (*n* = 4). Surgeries were performed by an ECVO diplomate in 7/30 (23%) dogs, by ECVO residents in 11/30 (37%), and by assistant veterinarians in 12/30 (40%). The surgeon group was not associated with a significant risk for developing SSI (Table 5). Only absorbable braided suture material was used for skin closure; therefore, no statistical comparison was performed.

#### 3.4.3 Antibiotic‐Related Risk Factors

**FIGURE 6:**
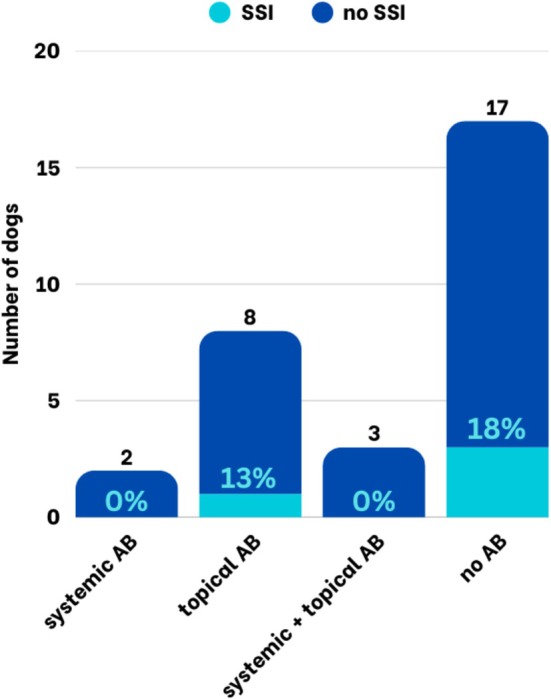
Proportion of dogs with and without SSI after simple eyelid surgeries considering different postoperative antibiotic regimens.

Postoperative antibiotics were administered to 13/30 (43%) patients, while 17/30 (57%) did not receive antibiotics. In dogs undergoing simple eyelid surgeries, the use of postoperative antibiotics had no significant effect on the occurrence of SSI. SSI occurred in 1/13 dogs receiving postoperative antibiotics (7.7%) and in 3/17 dogs without postoperative antibiotics (17.6%). Differentiation by regimen showed no significant effect: No SSI occurred in dogs receiving systemic or combined systemic–topical antibiotics, while SSI was observed in 12.5% of dogs receiving topical antibiotics and in 17.6% of those without postoperative antibiotics (Figure 6). The difference was not significant. The use of perioperative antibiotics, including the specific agents administered, was not significantly associated with the occurrence of SSIs (Table 5).

#### 3.4.4 Surgical Site Infections (SSI)

Overall, SSI occurred in 4/30 (13.3%) dogs (Table 7). Complications included purulent wound secretion (*n* = 3) and wound dehiscence (*n* = 3), with some dogs presenting both findings. According to the Clavien–Dindo classification, three cases were classified as grade 2, requiring a change in medical treatment. In one additional case, the exact grade could not be determined due to incomplete information from medical records and the owner. In one patient, a bacteriological culture was performed but yielded no bacterial growth.

### 3.5 Complicated Eyelid Surgeries

#### 3.5.1 Patient‐Related Risk Factors

A total of 22 dogs met the inclusion criteria. The mean age of the dogs was 2.0 ± 2.6 years (median 1 year, range 0–9 years; *n* = 22). Of the 22 dogs, 2 (9%) were classified as brachycephalic, 7 (32%) as giant breeds, and 5 (23%) had breed‐related heavy skin folds. The most common breeds were Great Danes (3/21; 14%) and mixed‐breed dogs (3/21; 14%). Shar Peis, Dogo Canarios, and Golden Retrievers were represented twice each (2/21; 10%). All other breeds were represented by a single individual. Dogs weighing 20–30 kg had a significantly lower SSI rate, while dogs weighing > 30 kg had a significantly higher SSI rate, whereas no significant difference was observed in dogs weighing 10–20 kg (Table 6). The primary indication for surgery in this group was entropion, either alone or in combination with other eyelid conformations (20/22; 91%), followed by ectropion (6/22; 27%) and macroblepharon (5/22; 23%). All other investigated patient‐related risk factors, including age, specific phenotypes, co‐morbidities and the influence of immunosuppression showed no significant association with SSI (Table 6).

**TABLE 6:** Risk factors for SSI after complicated eyelid surgeries.

Complicated eyelid surgeries (*n* = 22)				
Variables	Total (*n*)	SSI (*n*)	SSI (%)	*p*
*Weight (kg)*				
10–20	2	1	50.0	0.59*
20–30	5	0	0	**0.044** *
> 30	15	8	53.3	**0.049** *
*Phenotype*				
None	9	3	33.3	0.69*
Brachycephalic	2	1	50.0	1.00*
Giant breed (> 40 kg)	7	4	57.1	0.41*
Heavy breed related folds	5	2	40.0	1.00*
*Co‐morbidities*				
None	13	6	46.2	0.67*
Hypothyreoidism	1	0	0	1.00*
Skin lesions	4	2	50.0	1.00*
Other (non‐wound‐healing related)	4	1	25.0	1.00*
*Immunosuppression*				
No	22	9	40.9	
*Indication für surgery*				
Entropion	15	6	40.0	1.00*
Ectropion	1	0	0	1.00*
Mixed complicated (entropion and ectropion)	5	2	40.0	1.00*
Macroblepharon	5	2	40.0	1.00*
*Surgical approach*				
Modified Kuhnt–Szymanowski procedure combined with Celsus‐Hotz	14	5	35.7	0.41*
Gutbrod and Tietz technique	3	2	66.7	0.28*
Arrowhead technique	3	1	33.3	1.00*
Stades' forced granulation procedure	1	1	100.0	0.41*
Combinations of different surgical techniques	1	0	0	1.00*
*Surgeon group*				
Diplomate ECVO	5	2	40.0	
Resident ECVO	17	7	41.2	1.00*
*Perioperative antibiotics*				
Yes	21	9	42.9	
No	1	0	0	1.00*
*Perioperative antibiotics—agents*				
None	1	0	0	1.00*
Cefazolin/Cephalexin i.v. (1st‐generation cephalosporin) i.v.	10	6	60.0	0.20*
Cefuroxime i.v. (2nd‐generation cephalosporin) i.v.	8	2	25.0	0.40*
Amoxicillin–clavulanic acid (175 mg/mL) s.c.	3	1	33.3	1.00*
*Postoperative antibiotics*				
No antibiotics	15	6	40.0	
Systemic antibiotics	7	3	42.9	1.00*
*Postoperative antibiotics—agents*				
None	15	6	40.0	1.00*
Cefazolin/Cephalexin (1st‐generation cephalosporin) p.o.	4	2	50.0	1.00*
Amoxicillin–clavulanic acid p.o.	3	1	33.3	1.00*
*Suture material for skin closure*				
Absorbable braided	18	8	44.4	
Absorbabale monofilament	4	1	25.0	0.62*

*Note:* n/a from medical records; *p* < 0.05 is considered significant; bold numbers indicate statistical significance (*p* < 0.005).

Abbreviations: i.v., intravenously; n/a, not applicable; p.o., per oral; s.c., subcutaneously.

* Fisher's exact test (2 × 2).

#### 3.5.2 Surgery‐Related Risk Factors

The majority of dogs underwent a modified Kuhnt–Szymanowski and Celsus–Hotz correction (14/22; 64%), followed by the Gutbrod–Tietz technique (3/22; 14%) and the arrowhead technique (3/22; 14%). In addition, one dog (5%) was treated using Stades' forced granulation technique and one dog (5%) with a mixed technique. Surgeries were performed by an ECVO diplomate in 5/22 (23%) dogs *and* by ECVO residents in 17/22 (77%). The surgical approach, the surgeon group, and the suture material used for skin closure were not associated with a significant risk for developing SSI (Table 6).

#### 3.5.3 Antibiotic‐Related Risk Factors

**FIGURE 7:**
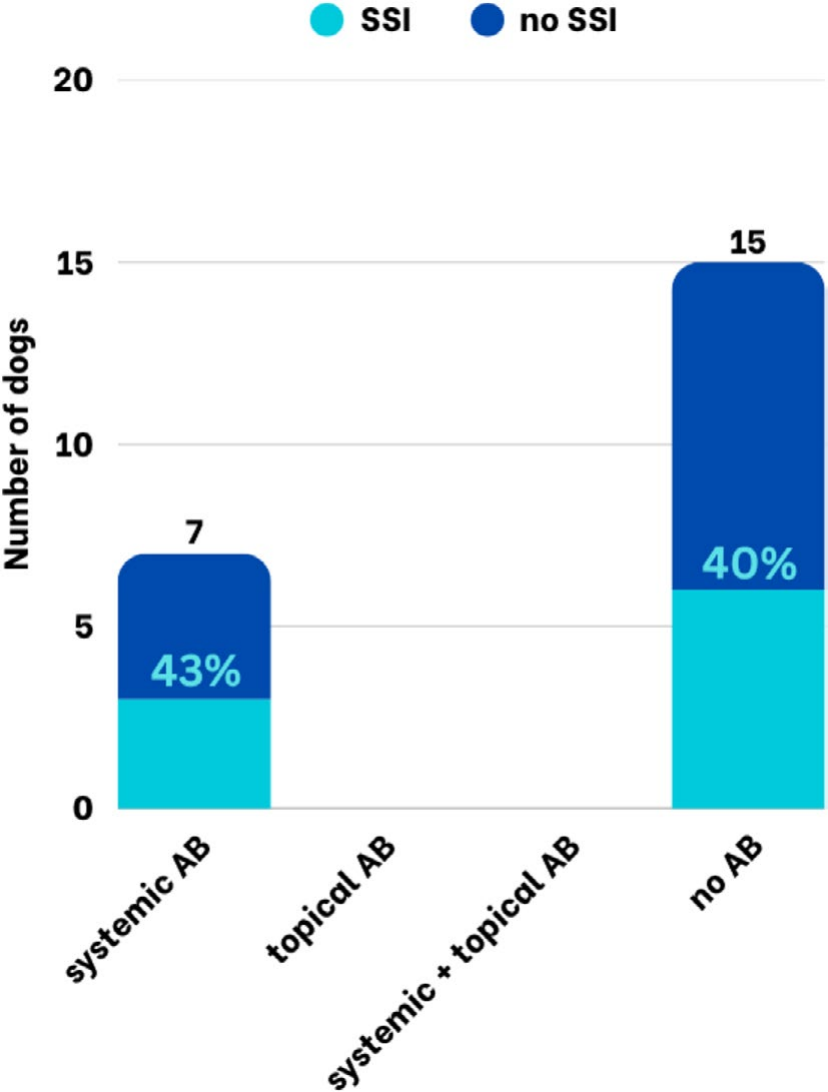
Proportion of dogs with and without SSI after complicated eyelid corrections considering different postoperative antibiotic regimens.

Postoperative antibiotics were administered to 7/22 (32%) patients, while 15/22 (68%) did not receive antibiotics. In dogs undergoing complicated eyelid surgeries, the use of postoperative antibiotics had no significant effect on SSI occurrence. SSI developed in 3/7 dogs receiving postoperative antibiotics (42.9%) and in 6/15 dogs without postoperative antibiotics (40.0%). Differentiation by regimen showed no significant differences: SSI occurred in 42.9% of dogs receiving systemic antibiotics and 40.0% of those without postoperative antibiotics, while no dogs received topical‐only or combined regimens (Figure 7). The difference was not significant. The use of perioperative antibiotics, including the specific agents administered, was not significantly associated with the occurrence of SSIs (Table 6).

#### 3.5.4 Surgical Site Infections (SSI)

Overall, SSI occurred in 9/22 (41%) dogs (Table 7). Complications included mucoid wound secretion (*n* = 2), purulent wound secretion (*n* = 1), and a combination of purulent wound secretion with wound dehiscence (*n* = 6). According to the Clavien–Dindo classification, eight cases were classified as grade 2, requiring a change in medical treatment, and one case as grade 1, in which no change in treatment was necessary.

**TABLE 7:** Distribution of postoperative complications according to Clavien–Dindo's Classification by surgical procedure.

Clavien–Dindo classification	Enucleations	Eyelid wedge resections	Medial canthoplasties	Simple eyelid surgeries	Complicated eyelid surgeries
Grade 1	3	0	2	0	1
Grade 2	1	7	1	3	8
Grade 3a	0	0	0	0	0
Grade 3b	1	1	2	0	0
Grade 4	0	0	0	0	0
Not documented	0	1	0	1	0

## 4 Discussion

Ophthalmic soft tissue surgeries, such as enucleations, eyelid wedge resections, and eyelid corrective procedures, represent routine interventions in the daily practice of both veterinary ophthalmologists and general practitioners. The objective of this study was to evaluate the correlation between the administration of postoperative antibiotics and the incidence of SSI after the procedures mentioned above. As our data has shown, there was no significant difference in SSI rates between patients who received postoperative antibiotics and those who did not in any of the evaluated ophthalmic soft tissue surgeries.

Accurately identifying and documenting SSIs is a critical step toward improving long‐term surgical outcomes and patient welfare. Over the past decade, there has been a growing body of research focused on the influence of antibiotics on SSI rates across various surgical procedures. In small animals, the SSI rate following minimally invasive abdominal or thoracic surgery has been reported at 1.7%, compared to 5.5% for those undergoing open approaches such as laparotomy or thoracotomy [22]. In orthopedic procedures, tibial plateau leveling osteotomy for cranial cruciate ligament rupture was associated with a higher SSI rate (8.4%) than extracapsular lateral suture stabilization (4.2%) [23]. There are only sparse publications about SSI after ophthalmic surgeries in veterinary medicine. A recent study reported an SSI incidence of 5% following enucleation in dogs [13]. In the referenced study, 3 out of 5 patients (60%) with SSI grew multidrug‐resistant organisms in their culture, which would not have been addressed by routine postoperative antibiotic administration. In a recent study by Allgoewer et al. SSI occurred in 2.1% of dogs, cats, and rabbits following a modified lateral enucleation technique, despite all patients receiving postoperative broad‐spectrum antibiotics [24]. Orbital cellulitis occurred as a postoperative complication in 5.1% of dogs that underwent enucleation with orbital implant placement [25]. In comparison, our findings showed a lower SSI rate, with 1.7% in dogs that did not receive postoperative antibiotics and 2.9% in those that did after enucleation surgeries. It should be noted that evisceration procedures with orbital implant placement were not included in the present study; however, a small number of dogs received an orbital mesh. To our knowledge, SSI rates for other veterinary ophthalmic surgeries have not been published yet.

Since our results indicate that postoperative antibiotic administration alone was not the determining factor for the development of SSI, other variables must contribute to the occurrence of SSI. In human medicine, a range of variables has been shown to be associated with the development of SSI. These include patient age, American Society of Anesthesiologists (ASA) status, duration of surgery and anesthesia, intraoperative hypotension, the number of people in the operating room, peri‐ and postoperative antibiotic administration, the presence of endocrine disorders, and the type of suture material used. In veterinary medicine, fewer studies are available; however, one study reported that the outcome “infection/inflammation” was significantly associated with several factors, including duration of surgery and anesthesia, an increasing number of people in the operating room, dirty surgical site classification, duration of postoperative intensive care unit stay, wound drainage, increasing patient weight, and antimicrobial prophylaxis [26, 27, 28, 29, 30, 31, 32, 33, 34]. In our study, the incidence of SSI following eyelid wedge resection was significantly higher in brachycephalic dog breeds and significantly lower in dogs without a distinct breed‐related phenotype, such as giant breeds or breeds characterized by pronounced skin folds. One possible explanation is that the periocular skin and skin folds in these breeds often harbor resistant bacteria and are prone to skin fold dermatitis [35], which may increase the risk of secondary infections in eyelid wounds. In our study, large‐breed dogs undergoing simple and complicated eyelid surgeries showed a significantly higher risk of SSI compared to smaller dogs, suggesting that body size may be a critical patient‐related risk factor in this procedure. In canine orthopedic procedures such as tibial plateau leveling osteotomy (TPLO), increased body weight has also been identified as a significant risk factor for SSI and other postoperative complications [23, 36] ‐ such an association has not yet been described in veterinary ophthalmology. It must be mentioned that only the body weight of the animals was recorded. A body condition score would have been more appropriate to determine whether the increased risk of SSI was associated solely with large breed size or also with overweight.

In human medicine, various classification systems for SSIs exist, enabling objective assessment and facilitating direct comparison between studies. As a veterinary‐specific system has yet to be established, adapted human classifications are currently used in veterinary settings. In our study, we applied the Clavien–Dindo classification of surgical complications [20], as it has been previously utilized in veterinary literature to address post‐operative complications, including SSI [37, 38]. The development of a standardized grading system for ophthalmic surgeries in veterinary medicine is strongly recommended and represents a future research topic. This would help to classify better and compare surgical procedures, as well as guide clinical decision‐making.

As mentioned above, in an online survey, two‐thirds of veterinarians reported using postoperative antibiotics always or most of the time following ophthalmic soft tissue procedures [10]. If we aim to change the current regimen, we must first understand the reasons behind it. The main reasons cited for this frequent use of systemic antibiotics were a fear of potential complications, personal experience, and diagnostic uncertainty [10]. This highlights a discrepancy, as our study demonstrates no association between postoperative antibiotic use and a reduced incidence of SSIs, suggesting that subjective concerns and personal experience often outweigh scientific evidence. Most of the participants (72%) were not or mostly not concerned that antimicrobial resistance may become a problem [10]. This perception fails to reflect the fact that the global rise in antimicrobial resistance is recognized as one of the most urgent public health threats worldwide [39]. Such denial or underestimation of established scientific evidence is highly concerning, as similar patterns of science denial are known from other critical areas, such as climate change [40]. The unnecessary use of antibiotics in a single patient contributes to the global burden of antimicrobial resistance and undermines the efficacy of future treatments. As resistance continues to rise across all classes of existing antimicrobials, the development of new antibiotics struggles to keep pace [41, 42, 43]. Rather than relying on antibiotics, we should improve our surgical outcomes by focusing on meticulous aseptic techniques, well‐structured perioperative protocols, and thorough postoperative wound care. Especially after surgeries affecting the eyelid margin, local wound management might be even more effective due to high drug concentrations that can be achieved at the application site and a lower incidence of systemic side effects [44]. Postoperative wound care can be achieved through the use of antiseptics, which offer a broader spectrum of antimicrobial activity than antibiotics [45].

When SSI occur, our data indicate that they are typically mild and manageable without the need for surgical reintervention in most cases. 81% of all SSIs in our study were classified as grade 1 or 2 according to Clavien–Dindo's classification of surgical complications, meaning infection control was achieved through a wait‐and‐see approach or a shift in medical management alone. Revision surgery was required in only 12.5% of patients with SSI, with equal distribution between those who received postoperative antibiotics and those who did not, indicating no apparent protective effect of postoperative antibiotic use.

This study has several limitations that should be taken into consideration. Due to its retrospective design, some medical records were incomplete, limiting the available data. The number of patients undergoing medial canthoplasties and eyelid corrective surgeries was relatively low, which affects the strength of conclusions for these subgroups of surgeries. One reason for the relatively small number of patients in this study is our inclusion criterion of a minimum 3‐month follow‐up, based on recent studies in human medicine that demonstrate a substantial proportion of SSIs occur beyond the traditional 30‐day surveillance period [46].

Bacteriological examinations were rarely performed, resulting in most SSIs being diagnosed clinically without microbiological confirmation. Nevertheless, it must be taken into consideration that wound culture may not always be a reliable diagnostic tool, as superficial wounds are frequently colonized by bacteria, which does not necessarily indicate true infection. We also had to rely on owners to provide accurate information regarding medication administration and the occurrence of SSIs, which introduces the risk of reporting bias. In individual cases, antibiotics were initiated by the referring veterinarian due to hematoma formation after enucleation surgery. While hematoma formation may represent a risk factor for SSIs due to blood serving as a favorable medium for bacterial growth, its presence is not automatically indicative of an existing SSI, and prescribing postoperative antibiotics might not have been necessary in these cases. However, these cases were classified as SSI according to Clavien–Dindo guidelines for surgical complications. Similarly, tension‐associated wound dehiscence, for example following medial canthoplasty procedures, and suture reactions may also have been misclassified as SSIs. Both conditions can clinically mimic infection due to swelling, erythema, discharge, or delayed healing, although they may represent non‐infectious postoperative complications. As our classification was partly based on the need for medical intervention (e.g., initiation of antibiotic therapy), it is possible that some of these cases were counted as SSIs despite not representing true infections per se. This leads to the next important limitation of our study. The lack of uniform definitions for SSIs in the veterinary literature further complicates interpretation. As a consequence, it is possible that some of the cases classified as SSI in our study may not have represented true infections per se. Distinguishing between normal postoperative inflammation, non‐infectious healing complications (such as hematoma or seroma), and a genuine SSI can be challenging. The use of standardized definitions and diagnostic criteria may help to reduce this limitation in future studies. Recently, a study aiming to establish more consistent criteria for SSI classification has been published and may provide valuable guidance for future research in this field [47].

The overall sample size and the number of SSI events were limited. Given the small number of outcome events and the presence of sparse data with small subgroup sizes, multivariable logistic regression was not performed, as this would have likely resulted in model instability and overfitting. Methodological recommendations suggest an adequate number of outcome events per variable to ensure reliable logistic modeling, which was not met in the present dataset. Therefore, the analysis was restricted to univariable comparisons using Fisher's exact test, which is appropriate for small sample sizes and low expected cell counts.

## 5 Conclusion

Postoperative antibiotics did not reduce the incidence of SSI in ophthalmic soft tissue surgeries. Given the global challenge of antimicrobial resistance, their routine prophylactic use should be critically questioned. The relatively high SSI rates observed after medial canthoplasties and eyelid surgeries emphasize the importance of optimizing pre‐, peri‐, and postoperative protocols, particularly with respect to proper wound hygiene practices.

## Author Contributions

**Ana Cristina Piroth:** conceptualization, investigation, writing – original draft, methodology, visualization, project administration, formal analysis. **Claudia Busse:** conceptualization, methodology, writing – review and editing.

## Disclosure

*AI Statement*: The authors have not used AI to generate any part of the manuscript.

## Ethics Statement

This study complies with the Guidelines for Ethical Research in Veterinary Ophthalmology (GERVO) and is exempt from approval by an ethics committee. Animal owners or owners' representatives provided written consent for the treatment provided.

## Conflicts of Interest

The authors declare no conflicts of interest.

## Data Availability

The data that support the findings of this study are available on request from the corresponding author. The data are not publicly available due to privacy or ethical restrictions.
